# Post-Operative Concurrent Chemoradiation for Patients with Non-Squamous Cell Carcinoma of Head and Neck: A Retrospective Cohort of the Uncommon Cancers

**DOI:** 10.31557/APJCP.2019.20.6.1727

**Published:** 2019

**Authors:** Lucksamon Thamlikitkul, Janjira Petsuksiri, Suthinee Ithimakin

**Affiliations:** 1 *Division of Medical Oncology, Department of Medicine, *; 2 *Division of Radiation Oncology, Department of Radiology, Faculty of Medicine, Siriraj Hospital, Mahidol University, Bangkok, Thailand. *

**Keywords:** Head and neck cancer, non-squamous carcinoma, salivary gland tumor, adjuvant, chemoradiation

## Abstract

**Background:**

Non-squamous cell carcinoma of the head and neck (HNnSCCA) is a rare tumor. Surgery is the standard treatment for resectable non-metastatic patients. Post-operative radiation (RT) is indicated for high-risk patients. No data from the randomized controlled trial utilizing post-operative concurrent chemoradiation (CCRT) is available. This study was aimed to determine the benefit of post-operative CCRT in the patients with resectable non-metastatic HNnSCCA.

**Methods:**

We retrospectively reviewed data of 139 patients with HNnSCCA (excluding nasopharyngeal, neuroendocrine, and skin cancers) who underwent surgery and post-operative radiation (RT) at Siriraj Hospital from 2009–2015.

**Results:**

Ninety-nine of the 139 patients had RT alone and 40 had CCRT. More patients receiving CCRT had ≥ one high-risk feature (80% CCRT vs. 57.6% RT; p=0.018). Five-year disease-free survival (DFS) and overall survival (OS) did not differ between the groups (58.6% CCRT vs. 68.2% RT; p=0.35 and 81.7% CCRT vs. 81.0% RT; p=0.35, respectively). Interestingly, post-operative CCRT was independently associated with significantly superior DFS (hazard ratio, HR 0.29; 95% confidence interval, CI 0.10 to 0.86; p=0.02) and OS (HR 0.08; 95% CI 0.01 to 0.43; p=0.003) according to multivariable analyses.

**Conclusion:**

Post-operative CCRT was associated with better survival in high-risk patients with resectable non-metastatic HNnSCCA comparing with post-operative RT alone. Post-operative CCRT might be considered as a treatment option for these patients.

## Introduction

*Background*

Malignancy of the head and neck is the ninth most common cancer worldwide (Gupta et al., 2016), thus representing a notable health burden. Squamous cell cancer is the most prevalent histologic type, being diagnosed in more than 90% of patients. Although non-squamous cancers in this anatomic region are rare, they present a significant controversial problem in oncological practice. Adenoid cystic carcinoma and mucoepidermoid carcinoma of major salivary glands are the two largest subgroups of non-squamous carcinoma (Guzzo et al., 2010).

Numerous Phase 3 clinical studies have strongly established standard treatments for head and neck squamous cell carcinoma (HNSCCA) and nasopharyngeal carcinoma (National Comprehensive Cancer Network, 2017). Patients with HNSCCA with high-risk features gain additional survival benefit from post-operative concurrent chemoradiotherapy (CCRT) over that achieved with radiotherapy (RT) alone (Bernier et al., 2004; Cooper et al., 2004; Bernier et al., 2005). Hence, administration of chemotherapy concurrently with RT has become the standard post-operative treatment for such patients. Definitive CCRT followed by adjuvant chemotherapy is thus standard treatment for non-metastatic, locally advanced nasopharyngeal cancer (Al-Sarraf et al., 1998). However, there are few data concerning appropriate therapeutic options for the remaining categories of head and neck non-squamous cell carcinoma (HNnSCCA). Consequently, oncologists usually base their management decisions on the data for HNSCCA (Cerda et al., 2014). Surgery remains the mainstay of treatment for patients with operable non-metastatic disease. Patients with high-risk features, including high histological grade or poor differentiation, advanced stage, perineural invasion, extracapsular lymph node extension and positive surgical margin (Pires et al., 2004; McHugh et al., 2012) may proceed to post-operative RT or CCRT with the aim of achieving maximum disease control and increasing the chance of cure based on potential radiation sensitizer effect of chemotherapy. However, combining chemotherapy with RT is associated with additional adverse effects, including severe mucositis, vomiting, renal impairment, and bone marrow suppression. 

To our knowledge, there is no consensus from results of randomized controlled trials (RCT) that post-operative CCRT offers survival benefits over RT alone in patients with HNnSCCA. The objective of this study was to assess the benefit of using chemotherapy concurrently with RT in patients with HNnSCCA.

## Materials and Methods

Electronic medical records of patients with head and neck cancer who had received post-operative RT at the Division of Radiation Oncology, Department of Radiology, Faculty of Medicine Siriraj Hospital, Mahidol University from 2006 to 2015 were retrospectively reviewed. The study cohort comprised patients with non-metastatic HNnSCCA who had undergone gross tumor removal and had no macroscopic residual disease according to either post-operative imaging or endoscopy. Nasopharyngeal cancer, neuroendocrine carcinoma and skin cancer were excluded because they require different oncologic management. Data from all recruited patients, including patient characteristics, initial stage, pathological features, concomitant chemotherapy use, radiotherapy interruption, date of recurrence and/or death, and sites of recurrence, were recorded, after which the oncological outcomes of those who received CCRT versus those who received RT alone were compared. Decisions to administer concomitant chemotherapy had been made at the medical oncologist and radiation oncologist’s discretion on an individual basis. The study was approved by Siriraj Institutional Review Board (Protocol number 836/2559 (EC3)).

The seventh edition of the American Joint Committee on Cancer’s staging manual was used to categorize tumor (T), nodal (N), and metastasis (M) stages and Stages I to IV. Because this staging manual does not cover some subsites of head and neck cancer (i.e. orbit and lacrimal glands), these cancers were classified as of undetermined stage. Additionally, because pathological criteria for tumor differentiation are not uniform for these rare tumors, some pathologists did not include this information in their reports. Such tumors were classified as being of undetermined differentiation.

The primary outcome was disease-free survival (DFS), defined as the duration from surgery until disease recurrence or death. The secondary outcomes included overall survival (OS), which was defined as the duration from surgery until death from any cause, patterns of recurrence, and compliance with treatment.

Baseline characteristics are presented as mean and standard deviation (SD) for continuous variables and number and percentage for categorical variables. The Mann–Whitney U test and Pearson’s χ^2^ or Fisher’s exact test were used to compare median and proportion, respectively. DFS and OS analyses were performed using the Kaplan–Meier method and comparisons were made by log-rank test. Simple and multiple Cox regression analyses were used to assess predictors of disease recurrence and death. Statistical analyses were carried out using PASW Statistics 18.0, Chicago, IL, USA. A p-value of less than 0.05 was considered to denote statistical significance.

## Results

**Figure 1:**
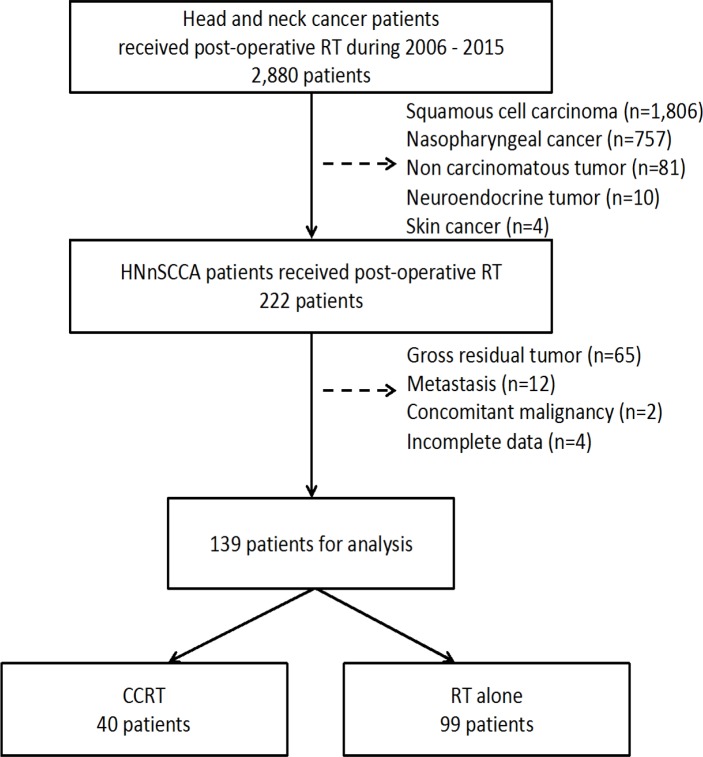
Flow Chart Showing Patient Selection

We identified 2,880 patients with head and neck cancer who had received post-operative RT at Siriraj Hospital from January 2006 to December 2015. We excluded 2,658 patients with squamous cell carcinoma, nasopharyngeal cancer, neuroendocrine carcinoma, skin cancer and non-carcinoma histology. After also excluding patients with gross residual disease, metastatic disease, and concomitant malignancy, 139 patients remained for analysis (Figure 1). We divided these patients into two groups: RT alone (n=99, 71%) and CCRT (n=40, 29%). 

Baseline patient characteristics are shown in Table 1. The mean age, sex distribution, histology, and primary site did not differ significantly between the CCRT and RT groups. Adenoid cystic carcinoma was the most common HNnSCCA (35%), followed by mucoepidermoid carcinoma (24%). Most of the HNnSCCAs originated from major salivary glands (68%). There was a significantly higher proportion of patients with higher clinical and nodal stage, poor tumor differentiation, extranodular extension, and lymphovascular/perineural invasion in the CCRT group. In the CCRT group, three patients (7.5%) had Stage I disease and 15 (37.5%) Stage IV disease, whereas in the RT group 27 patients (27.3%) had Stage I disease and 13 (13.1%) Stage IV disease (p = 0.004). Mean tumor size was greater in the CCRT group (3.4 cm vs. 3.0 cm; p = 0.045). More patients in the CCRT group had N2 disease (27.5% vs. 4%; p < 0.001). Poor tumor differentiation was more common in the CCRT group (32.5% vs. 6.1%; p < 0.001). Extranodular extension was present in 27.5% and 2% of the CCRT and RT groups, respectively (p = 0.011). Lymphovascular and perineural invasion were more frequently observed in the CCRT group (27.5% vs. 12.1%; p = 0.027 and 52.5% vs. 31.3%, respectively; p = 0.019,). Overall, a majority of patients in both groups had at least one high-risk feature. However, the proportion of patients with at least one high-risk feature was significantly higher in the CCRT than the RT alone group (80% vs. 57.6%; p = 0.018).

*Disease-free survival (DFS)*

**Figure 2:**
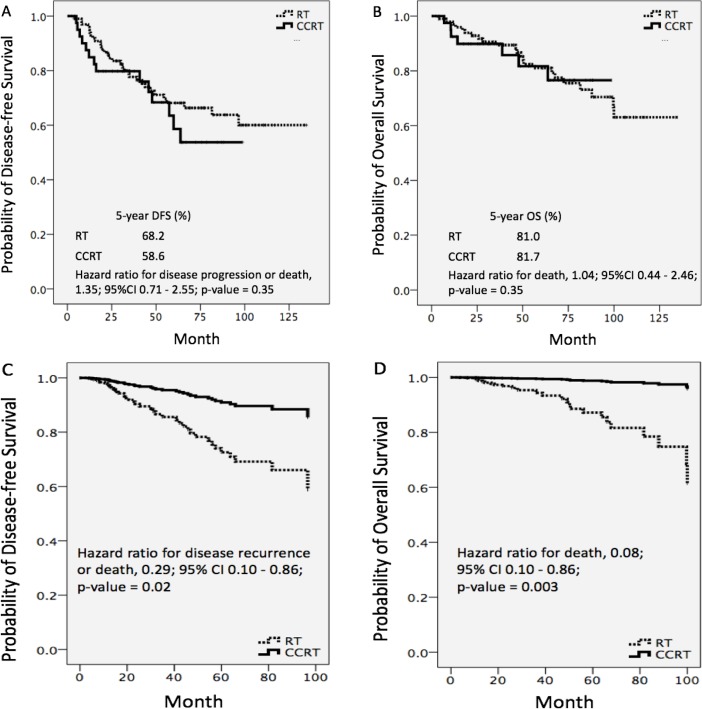
Unadjusted Disease-free Survival (A); Unadjusted Overall Survival (B); Multivariable-adjusted Disease-free Survival (C); and Overall Survival (D) According to Treatment Group

At the median follow-up time of 58.8 months, disease recurrence or death had occurred in 14 patients (35%) in the CCRT and 31 patients (31.3%) in the RT group. Median DFS was not reached and did not differ significantly between the two groups. The estimated proportion of patients who were alive without disease recurrence at 5 years was 58.6% and 68.2% in patients who had received CCRT and RT, respectively. The 5-year DFS rate did not differ between the two groups (hazard ratio 1.35; 95% confidence interval 0.71 to 2.55; p = 0.35) (Figure 2A). To compensate for the unequal distribution of prognostic factors between groups, we performed univariable and multivariable analyses, adjusting for multiple known prognostic factors (Table 2). According to multivariable analysis, CCRT was significantly associated with a 71% lower risk of disease progression or death than RT alone (HR 0.29; 95% CI 0.10 to 0.86; p = 0.02). The Kaplan–Meier DFS curves differed distinctly between the CCRT and RT groups (Figure 2C). Patients whose tumors had originated in the oral cavity, larynx, or paranasal sinus had a greater risk of tumor recurrence or death than those whose tumors had originated in a major salivary gland. Poor tumor differentiation and perineural invasion were also associated with shorter DFS.

*Overall survival (OS)*

At the median follow-up time of 58.8 months, seven patients (17.5%) in the CCRT and 23 (23.2%) in the RT group had died. Median OS was not reached in either group. We estimated that 81.7% and 81.0% of patients with CCRT and RT alone, respectively, were alive at 5 years with an HR of 1.04 (95% CI 0.44–2.46; p = 0.35) (Figure 2B). The duration of OS was not different between the groups. Results of univariable and multivariable analyses for predictors of death are shown in Table 3. After adjusting for multiple prognostic factors, we found that CCRT was significantly associated with a 92% lower risk of death than RT (HR 0.08; 95% CI 0.01 to 0.43; p = 0.003). We again noted obvious differences between the Kaplan–Meier OS curves for the RT and CCRT groups (Figure 2D). As to tumor subsites, patients with HNnSCCA whose tumors were located in the oral cavity, oropharynx, or larynx had shorter OS than those with tumors in a major salivary gland. Tumor size larger than three centimeters and poor differentiation were adverse prognostic factors for OS.

*Pattern of recurrence*

Disease recurrence occurred in 11 (27.5%) and 24 patients (24.2%) in the CCRT and RT groups, respectively; this difference was not statistically significant (p = 0.19). A higher proportion of patients receiving postoperative CCRT than RT alone developed locoregional recurrences (10% vs. 4%, p = 0.22). Distant metastasis occurred in seven patients (17.5%) in the CCRT and 20 (20.2%) in the RT group (p = 0.81). The most common sites of distant metastasis were lung and bone.

*Compliance with treatment*

Compliance with radiotherapy was excellent in both groups. Mean (SD) radiation dose was 63.4 (3.4) Gy and 62.6 (5.7) Gy in the CCRT and RT groups, respectively (p = 0.87). The radiotherapy schedule was interrupted in five patients (12.5%) in the CCRT and six (6.1%) in the RT group (p = 0.29). Only one patient (1%) in the RT group did not complete the planned radiation schedule, whereas all other 138 patients in both groups did. 

Thirty-seven patients (92.5%) in the CCRT group received three-weekly cisplatin whereas three (7.5%) received weekly carboplatin as a radio-sensitizing agent.

**Table 1:** Baseline Patient Characteristics

	CCRT (n=40)	RT alone (n=99)	p-value
Male, n (%)	19 (47.5)	41 (41.4)	0.57
Mean age (SD) (years)	48.4 (14.7)	51.5 (15.2)	0.36
Tumor site, n (%)			
Major salivary gland	23 (57.5)	71 (71.7)	0.09
Paranasal sinus	5 (12.5)	5 (5.1)	
Nasal cavity	2 (5)	8 (8.1)	
Oral cavity	3 (7.5)	6 (6.1)	
Oropharynx	3 (7.5)	2 (2.0)	
Larynx	3 (7.5)	1 (1)	
Orbit and lacrimal glands	1 (2.5)	6 (6.1)	
Mean tumor size (SD) (cm)	3.39 (1.59)	3.03 (1.95)	0.045
Clinical stage, n (%)			
I	3 (7.5)	27 (27.3)	0.004
II	9 (22.5)	28 (28.3)	
III	12 (30.0)	24 (24.2)	
IV	15 (37.5)	13 (13.1)	
Undetermined	1 (2.5)	7 (7.1)	
Tumor stage, n (%)			
T1	4 (10.0)	27 (27.3)	0.14
T2	18 (45.0)	32 (32.3)	
T3	12 (30.0)	24 (24.2)	
T4	5 (12.5)	9 (9.1)	
Undetermined	1 (2.5)	7 (7.1)	
Nodal stage, n (%)			
N0	24 (60)	89 (89.9)	<0.001
N1	5 (12.5)	6 (6.1)	
N2	11 (27.5)	4 (4.0)	
Histology, n (%)			
Adenoid cystic carcinoma	14 (35)	35 (35.4)	0.30
Mucoepidermoid carcinoma	8 (20)	28 (28.3)	
Salivary duct carcinoma	5 (12.5)	5 (5.1)	
Acinic cell carcinoma	2 (5)	8 (8.1)	
Adenocarcinoma	2 (5)	8 (8.1)	
Lymphoepithelioma	4 (10)	3 (3)	
Carcinoma ex pleomorphic adenoma	0 (0)	5 (5.1)	
Basal cell carcinoma	1 (2.5)	1 (1)	
Other	4 (10)	6 (6.1)	
Tumor differentiation, n (%)			
Well	2 (5)	19 (19.2)	<0.001
Moderately	1 (2.5)	8 (8.1)	
Poorly	13 (32.5)	6 (6.1)	
Undetermined	24 (60)	66 (66.7)	
Margin status, n (%)			
R0	3 (7.5)	21 (21.2)	0.25
R1	20 (50)	38 (38.4)	
Close	12 (30)	29 (29.3)	
Gross tumor removal with			
undetermined microscopic margin	5 (12.5)	11 (11.1)	
Extranodular extension, n (%)			
Absent	35 (87.5)	97 (98)	0.011
Present	5 (12.5)	2 (2)	
Lymphovascular invasion, n (%)			
Absent	29 (72.5)	87 (87.9)	0.027
Present	11 (27.5)	12 (12.1)	
	CCRT (n=40)	RT alone (n=99)	p-value
Perineural invasion, n (%)			
Absent	19 (47.5)	68 (68.7)	0.019
Present	21 (52.5)	31 (31.3)	

RT, radiation; CCRT, concurrent chemoradiation; SD, standard deviation; R0, clear margin; R1, microscopic residual tumor; Close margin, distance from tumor to resected margin less than 0.5 cm

**Table 2:** Results of Univariable and Multivariable Analyses for Predictors of Disease-free Survival

	Crude HR [95% CI]	p-value	Adjusted HR [95% CI]	p-value
Tumor site		<0.001		<0.001
Major salivary gland	1		1	
Nasal cavity	1.29 [0.38, 4.28]	0.67	0.26 [0.02, 2.51]	0.24
Oral cavity	2.27 [0.78, 6.57]	0.13	5.46 [1.56, 19.15]	0.008
Oropharynx	1.81 [0.43, 7.69]	0.41	2.65 [0.49, 14.21]	0.25
Larynx	24.57 [7.50, 80.47]	<0.001	60.33 [11.61, 313.46]	<0.001
Paranasal sinus	3.98 [1.71, 9.28]	0.001	3.94 [1.24, 12.44]	0.01
Histology		0.01		0.45
ACC	1		1	
MEC	0.28 [0.10, 0.74]	0.01	0.48 [0.09, 2.54]	0.39
Salivary duct carcinoma	1.34 [0.51, 3.52]	0.55	1.27 [0.24, 6.49]	0.77
CEPA	1.05 [0.24, 4.51]	0.94	2.68 [0.48, 14.78]	0.25
Adenocarcinoma	1.56 [0.59, 4.16]	0.36	1.76 [0.42, 7.41]	0.43
Other	0.28 [0.09, 0.81]	0.02	0.50 [0.12, 2.08]	0.34
Tumor diameter		0.02		0.11
≤ 3 cm	1		1	
> 3 cm	2.03 [1.10, 3.77]	0.02	1.98 [0.85, 4.59]	0.11
Tumor differentiation		0.002		0.02
Well and moderately	1		1	
Poorly	29.57 [3.76, 232.19]	0.001	23.50 [2.30, 239.49]	0.008
Undetermined	12.33 [1.68, 90.24]	0.013	7.42 [0.71, 76.63]	0.09
Nodal stage		0.25		0.48
N0	1		1	
N1	1.76 [0.68, 4.50]	0.23	0.82 [0.18, 3.76]	0.80
N2	1.79 [0.75, 4.27]	0.18	2.15 [0.46, 10.06]	0.32
Lymphovascular invasion		0.02		0.34
Absent	1		1	
Present	2.24 [1.13, 4.45]	0.02	1.64 [0.58, 4.63]	0.34
Perineural invasion		0.001		0.04
Absent	1		1	
Present	2.68 [1.49, 4.85]	0.001	2.39 [1.02, 5.59]	0.04
Surgical margin		0.52		0.73
R0	1		1	
R1	1.82 [0.68, 4.83]	0.22	2.01 [0.57, 6.99]	0.27
Close margin	1.37 [0.48, 3.90]	0.54	1.46 [0.43, 4.85]	0.53
GTR	2.11 [0.66, 6.67]	0.20	1.74 [0.28, 10.90]	0.55
Treatment		0.35		0.02
Radiation alone	1		1	
Chemoradiation	1.35 [0.71, 2.55]	0.35	0.29 [0.10, 0.86]	0.02

HR, hazard ratio; CI, confidence interval; ACC, adenoid cystic carcinoma; MEC, mucoepidermoid carcinoma; CEPA, carcinoma ex pleomorphic adenoma; GTR, gross tumor removal with undetermined microscopic margin; R0, clear margin; R1, microscopic residual tumor; Close margin, distance from tumor to resected margin less than 0.5 cm

**Table 3:** Results of Univariable and Multivariable Analyses for Predictors of Overall Survival

	Crude HR [95% CI]	p-value	Adjusted HR [95% CI]	p-value
Tumor site		<0.001		<0.001
Major salivary gland	1		1	
Nasal cavity	1.98 [0.57, 6.81]	0.27	0.62 [0.05, 7.44]	0.71
Oral cavity	2.50 [0.72, 8.63]	0.14	21.23 [3.56, 126.64]	0.001
Oropharynx	3.39 [0.77, 14.85]	0.10	20.49 [2.59, 161.71]	0.004
Larynx	22.93 [5.95, 88.29]	<0.001	187.06 [22.09, 1583.6]	<0.001
Paranasal sinus	3.04 [0.87, 10.57]	0.08	4.97 [0.74, 32.98]	0.09
Histology		0.20		0.29
ACC	1		1	
MEC	0.67 [0.23, 1.91]	0.45	1.99 [0.24, 16.33]	0.52
Salivary duct carcinoma	2.10 [0.67, 6.54]	0.19	4.15 [0.52, 32.79]	0.17
CEPA	3.21 [0.69, 14.96]	0.13	11.76 [1.48, 93.38]	0.02
Adenocarcinoma	2.40 [0.65, 8.83]	0.18	4.66 [0.55, 39.34]	0.15
Other	0.75 [0.24, 2.37]	0.63	2.55 [0.43, 15.00]	0.30
Tumor size		0.02		0.02
≤ 3 cm	1		1	
> 3 cm	2.51 [1.15, 5.48]		3.8 [1.16, 12.38]	0.02
Tumor differentiation		0.002		0.005
Well and moderately	1		1	
Poorly	21.01 [2.60, 169.49]	0.004	64.42 [4.56, 908.20]	0.002
Unknown	6.59 [0.88, 49.04]	0.06	11.03 [0.65, 184.95]	0.09
Nodal stage		0.06		0.34
N0	1		1	
N1	2.40 [0.82, 7.02]	0.10	0.61 [0.09, 3.96]	0.61
N2	2.67 [0.99, 7.16]	0.05	2.71 [0.50, 14.53]	0.24
Lymphovascular invasion		0.32		0.43
Absent	1		1	
Present	1.56 [0.63, 3.84]	0.32	1.71 [0.43, 6.74]	0.43
Perineural invasion		0.41		0.27
Absent	1		1	
Present	1.35 [0.65, 2.79]	0.41	1.81 [0.62, 5.31]	0.27
Margin status		0.74		0.36
R0	1		1	
R1	1.37 [0.44, 4.20]	0.58	4.12 [0.76, 22.09]	0.09
Close	1.19 [0.35, 3.95]	0.77	3.62 [0.78, 16.76]	0.10
GTR	1.98 [0.52, 7.45]	0.31	4.96 [0.30, 79.89]	0.25
Treatment		0.92		0.003
Radiation alone	1		1	
Chemoradiation	1.04 [0.44, 2.46]	0.92	0.08 [0.01, 0.43]	0.003

HR, hazard ratio; CI, confidence interval; ACC, adenoid cystic carcinoma; MEC, mucoepidermoid carcinoma; CEPA, carcinoma ex pleomorphic adenoma; GTR, gross tumor removal with undetermined microscopic margin; R0, clear margin; R1, microscopic residual tumor; Close margin, distance from the tumor to the resected margin less than 0.5 cm

## Discussion

In this study, there was no difference in the duration of DFS and OS between patients who received post-operative CCRT and RT alone according to univariable analysis. Interestingly, multivariable analysis showed a significant positive independent association between CCRT administration and DFS and OS. Patients who received post-operative CCRT had a substantially lower risk of disease recurrence or death (HR 0.29; 95% CI 0.10 to 0.86; p = 0.02) and death (HR 0.08; 95% CI 0.01 to 0.43; p = 0.003) than those who received RT only. Unfortunately, there were insufficient patients in the subgroups to allow us to perform multivariable analyses to determine which subgroup(s) derived the greatest benefit from CCRT.

A significantly higher proportion of patients who received CCRT than of those who received RT alone had high-risk prognostic factors, including large tumor, high stage, poor differentiation, extranodular extension, and lymphovascular and perineural invasion. Thus, patients in CCRT group tended to have poorer prognoses to begin with, regardless of treatment. This imbalance of poor prognostic factors between the treatment groups may have contributed to our failure to detect a survival benefit for CCRT by univariable analysis. 

Post-operative RT was administered after resection of malignant salivary gland tumors with high-risk features (Safdieh et al., 2017), even though these tumors are relatively radioresistant (Cerda et al., 2014). Given that platinum-based chemotherapy reportedly enhances radiotherapy-induced DNA damage to tumor cells via various mechanisms (Marcu et al., 2003), many clinical trials have been performed to investigate this synergistic effect. We identified an additional survival benefit from combining chemotherapy with radiotherapy for patients with HNSCCA, as mentioned earlier. The low incidence of HNnSCCA means that it is not practicable to conduct large RCT on these patients. Multiple retrospective studies that have investigated the benefit of post-operative CCRT in patients with major salivary gland tumors have found no difference in survival between patients receiving postoperative CCRT versus RT alone (Tanvetyanon et al., 2009; Schoenfeld et al., 2012; Amini et al., 2016; Mifsud et al., 2016). Notwithstanding that, Tanvetyanon et al., (2009) reported a marginally greater three-year overall survival (44% vs. 83%) for patients receiving CCRT versus RT alone, respectively (p = 0.05). Also, multivariable analyses from those studies have not identified an association between CCRT and survival. Some dissimilarities between our study and those four studies are worthy of note. We included patients with non-squamous carcinomas of all head and neck subsites in our study, whereas previous studies have included only patients with major salivary gland tumors. Even though major salivary gland tumors comprise the majority of HNnSCCA; there is also a sizeable proportion of non-squamous carcinomas of other head and neck regions, for example, the nasal cavity, paranasal sinuses, oral cavity, and oropharynx. We considered it important to include this minority of patients to enhance the generalizability of our findings. Furthermore, we only included patients with no gross residual tumor after the surgery, this having been confirmed in all study patients by either post-operative imaging or endoscopy. Because the therapeutic dose of post-operative RT is different for patients with microscopic versus macroscopic disease, ensuring homogeneity of the study cohort required exclusion of patients with gross residual disease. The recommended RT dose for microscopic disease is 60–63 Gy (Peters et al., 1993) which is comparable to the mean RT dose administered to our patients. Another possible explanation for the favorable outcomes of CCRT in our study is the excellent compliance with both radiation and chemotherapy. Moreover, our median follow-up time was 58.8 months, which is longer than in prior studies. 

We acknowledge that our study has possible biases and limitations of all retrospective studies. Although there was heterogeneity in recruited population, the study provides information with the attempt to answer the argument in real-world clinical practice regarding management in these uncommon tumors. In addition, longer follow up may be needed to confirm survival data since there was substantial patients with long survival expectation. The results of the RCT are necessary to draw an unequivocal conclusion on the benefits of post-operative CCRT after resection of HNnSCCAs. RTOG 1008 is a current Phase II/III RCT evaluating overall survival after resection of high-risk malignant salivary gland tumors (The Radiation Therapy Oncology Group, 2017). Eligible participants are randomized to receive either concurrent cisplatin with RT or RT alone. This study is still recruiting patients and expected to be completed in October 2023. This trial will explicitly determine whether post-operative CCRT is beneficial in this group of patients. 

Concurrent CCRT is independently associated with better DFS and OS than RT alone in patients with high-risk non-metastatic HNnSCCA who have undergone gross tumor resection. Post-operative CCRT might be considered as a treatment option for these patients. Further results from larger randomized controlled study are awaited.
